# Self-Help Plus for refugee mothers in Rhino Refugee Settlement, Uganda (SEED): study protocol for a cluster-randomized controlled trial assessing intergenerational effects on preschool-aged children

**DOI:** 10.1186/s13063-026-09546-1

**Published:** 2026-02-17

**Authors:** Phaidon T. B. Vassiliou, Herbert E. Ainamani, Stefan Döring, Gustaf Gredebäck, Marx R. Leku, Kirsi Peltonen, Florian Scharpf, Umay Sen, Matthias Sutter, James Igoe Walsh, Tobias Hecker, Jonathan Hall

**Affiliations:** 1 Department of Peace and Conflict Research, Uppsala University, Uppsala, Sweden; 2 Department of Mental Health, Kabale University School of Medicine, Kabale University, Kabale, Uganda; 3 Peace Research Institute Oslo (PRIO), Oslo, Norway; 4 Centre for Impacts of Climate Extremes (climes), Uppsala University, Uppsala, Sweden; 5 Department of Psychology, Uppsala University, Uppsala, Sweden; 6 Muni University, Arua, Uganda; 7 INVEST Research Flagship Centre, University of Turku, Turku, Finland; 8 Department of Psychology, Bielefeld University, Bielefeld, Germany; 9 Institute for Interdisciplinary Conflict and Violence Research, Bielefeld University, Bielefeld, Germany; 10 Max Planck Institute for Research on Collective Goods, Bonn, Germany; 11 Department of Economics, University of Cologne, Cologne, Germany; 12 Department of Public Finance, University of Innsbruck, Innsbruck, Austria; 13 Department of Political Science, School of Data Science, and Program in Public Policy, University of North Carolina at Charlotte, Charlotte, USA

**Keywords:** Child development, Cluster-randomized controlled trial, Intergenerational effects, Mental health, Parenting, Refugees, Self‑Help Plus, Uganda

## Abstract

**Background:**

Growing up in adversity can create enduring deficits in children’s cognitive and socio-behavioral skills that undermine later-life productivity, reduce human capital, and increase social costs. Early interventions that target caregiver mental health offer a promising pathway to strengthen the developmental environment of children exposed to severe stress. Yet, in low-resource humanitarian settings, evidence on scalable approaches that generate such intergenerational benefits remains limited. War-related displacement places mothers and young children at exceptional risk for psychological distress and impaired functioning, with potential long-term consequences for both generations. *Self-Help Plus* (SH+), a brief, low-intensity WHO group intervention based on Acceptance and Commitment Therapy, has shown promising short-term effects in reducing psychological distress among South Sudanese refugee women in Rhino Camp, Uganda. However, key questions remain regarding the durability of these effects and whether improvements in maternal mental health translate into measurable gains in children’s own wellbeing and early development.

**Methods:**

This two-arm, parallel-group cluster-randomized controlled trial will enroll 720 mother-preschool-aged child (3–5 years) dyads from 24 villages in Rhino Refugee Settlement, Uganda. Villages are randomized 1:1 to receive either SH+ and Enhanced Usual Care (EUC), or EUC only. Assessments are conducted at baseline (T0), 3 months (T1), and 12 months (T2) post-intervention. The primary outcome is maternal psychological distress (Kessler-6) at 12 months (T2). The key secondary outcome is parent-reported child psychosocial wellbeing (Kiddy-KINDLR) at T2. Secondary outcomes include additional indicators of maternal wellbeing and mental health, parenting practices, and child outcomes assessed across study time points, including psychosocial difficulties and child self-reported wellbeing. Analyses will follow an intention-to-treat approach, adjusting for clustering and relevant covariates.

**Discussion:**

This trial replicates and extends prior evidence on SH+ in a large refugee population. It will examine whether early mental health gains are sustained, and whether intergenerational benefits emerge for preschool-aged children. Findings will inform scalable intervention strategies to promote psychological resilience and child development in humanitarian contexts.

**Trial registration:**

ClinicalTrials.gov NCT07062341. Prospectively registered on July 11, 2025.

**Supplementary Information:**

The online version contains supplementary material available at 10.1186/s13063-026-09546-1.

## Administrative information

Note: the numbers in curly brackets in this protocol refer to SPIRIT checklist item numbers. The order of the items has been modified to group similar items (see http://www.equator-network.org/reporting-guidelines/spirit-2013-statement-defining-standard-protocol-items-for-clinical-trials/).

**Table:** 

Title {1}	Self-Help Plus for refugee mothers in Rhino Camp Refugee Settlement, Uganda (SEED): study protocol for a cluster-randomized controlled trial assessing intergenerational effects on preschool-aged children
Trial registration {2a and 2b}	ClinicalTrials.gov identifier: NCT07062341. Prospectively registered on July 11, 2025.
Protocol version {3}	Version 2
Funding {4}	This study is supported by Riksbankens Jubileumsfond (grant no. P22-0514)
Author details {5a}	Phaidon T.B. Vassiliou^1^, Herbert E. Ainamani^2^, Stefan Döring^1,3,4^, Gustaf Gredebäck^5^, Marx R. Leku^6^, Kirsi Peltonen^7^, Florian Scharpf^8,9^, Umay Sen^5^, Matthias Sutter^10,11,12^, James Igoe Walsh^13^, Tobias Hecker^8,9,†^, Jonathan Hall^1,†^ 1. Department of Peace and Conflict Research, Uppsala University, Uppsala, Sweden 2. Department of Mental Health, Kabale University School of Medicine, Kabale University, Kabale, Uganda 3. Peace Research Institute Oslo (PRIO), Oslo, Norway 4. Centre for Impacts of Climate Extremes (climes), Uppsala University, Uppsala, Sweden 5. Department of Psychology, Uppsala University, Uppsala, Sweden 6. Muni University, Arua, Uganda 7. INVEST Research Flagship Centre, University of Turku, Turku, Finland 8. Department of Psychology, Bielefeld University, Bielefeld, Germany 9. Institute for Interdisciplinary Conflict and Violence Research, Bielefeld University, Bielefeld, Germany 10. Max Planck Institute for Research on Collective Goods, Bonn, Germany 11. Department of Economics, University of Cologne, Cologne, Germany 12. Department of Public Finance, University of Innsbruck, Innsbruck, Austria 13. Department of Political Science, School of Data Science, and Program in Public Policy, University of North Carolina at Charlotte, Charlotte, USA ^†^ These authors share senior authorship. Corresponding author: Jonathan Hall, Department of Peace and Conflict Research, Uppsala University, Uppsala, Sweden Email: jonathan.hall@pcr.uu.se
Name and contact information for the trial sponsor {5b}	This study is sponsored by Uppsala University. UCR-Uppsala Clinical Research Center info@ucr.uu.se Uppsala Science Park Dag Hammarskjölds väg 38 751 85 Uppsala (SE)
Role of sponsor {5c}	The sponsor and funder have had no role in the design or submission of this protocol. They will have no role in the collection, management, analysis, and interpretation of data.

## Introduction

### Background and rationale {6a}

Mental disorders contribute substantially to the global burden of disease and disability [1]. War-exposed refugee populations are at exceptionally high risk of mental health disorders, including post-traumatic stress disorder, anxiety, and depression [2, 3]. Refugee mothers, in particular, face distinct challenges that compound this risk, such as exposure to gender-based violence, the psychological burden of caregiving in precarious conditions, and systemic barriers to accessing support [4].

A mother’s psychological state is a cornerstone of the caregiving environment that fosters child development. Positive maternal mental health supports nurturing parenting and promotes children’s development and wellbeing [5, 6]. Conversely, psychological distress can destabilize family dynamics and reduce caregiving quality, hindering children’s socio-emotional and cognitive development [7–9]. These effects are especially consequential during the preschool years (ages 3–5), a period of heightened neuroplasticity when the developing brain is highly sensitive to environmental inputs [10].

These psychological burdens are particularly acute in humanitarian contexts, where exposure to past trauma combines with ongoing adversity. Yet a major treatment gap persists due to the scarcity of scalable, evidence-based interventions [11]. Beyond symptom reduction, improving maternal mental health may also strengthen caregiving and family functioning, with downstream implications for young children’s psychosocial wellbeing. Recent consensus-based research priorities for mental health and psychosocial support (MHPSS) in humanitarian settings highlight the need to assess both short-term and long-term benefits of MHPSS interventions and to better understand intergenerational impacts of adversity [11]. These priorities motivate the present trial’s 12-month endpoint and the inclusion of child psychosocial wellbeing as a key outcome. If efficient interventions can produce sustained improvements in maternal mental health, they may yield a double benefit: promoting maternal wellbeing while simultaneously fostering positive child development.

Self‑Help Plus (SH+), a five‑session, multimedia‑guided group program developed by the World Health Organization for delivery by trained lay providers [12, 13], offers a promising scalable solution. In a prior cluster‑randomized trial in Rhino Refugee Settlement, SH+ significantly reduced women’s psychological distress at 3 months compared to enhanced usual care [14]. Evidence from LMICs suggests that improving maternal mental health can yield some child-related benefits, but findings are mixed and often inconclusive across child outcomes, with few trials assessing longer-term child psychosocial and developmental endpoints [15]. Important questions remain regarding (i) whether these benefits endure over time and (ii) whether improvements in maternal mental health translate into measurable benefits for preschool-aged children’s psychosocial wellbeing and functioning [11].

A more integrated understanding of how conflict-related stressors intersect across psychological, family, and developmental domains is urgently needed to guide policy and programming in humanitarian settings [16]. This study contributes to this goal by examining whether enhancing maternal mental health can promote child development, providing critical evidence on the broader “spillover” effects and potential societal return on investment of scalable mental health interventions in resource-constrained, conflict-affected settings.

### Objectives {7}

The Self-Help Plus to Enhance Early Development (SEED) trial seeks to determine whether Self‑Help Plus (SH+) produces sustained mental‑health benefits for war-exposed displaced mothers and generates intergenerational gains for their preschool‑aged children. The primary outcome is maternal psychological distress (Kessler-6) at 12 months post-intervention (T2). The key secondary outcome is parent-reported child psychosocial wellbeing (Kiddy-KINDL^R^ Parent Report) at T2. Secondary outcomes include additional indicators of maternal wellbeing and mental health indicators, parenting practices, and child psychosocial outcomes assessed across study time points (including child self-reported wellbeing and psychosocial functioning). A range of exploratory outcomes, including socio-behavioral skills (patience, risk tolerance, and prosociality), cognitive development, social capital, household wellbeing (e.g., food security), and other ancillary domains, will be analyzed and reported in separate planned publications.

Primary hypothesis:

- H1—Maternal mental health: Mothers in SH+ villages will report lower psychological distress (Kessler‑6) [17] at 12 months than mothers in EUC villages.

Key secondary hypothesis:

- H2—Child well‑being: Children whose mothers receive SH+ will exhibit higher psychosocial well‑being (Kiddy‑KINDL^R^ - Parent Report) [18] at 12 months than children in EUC villages.

Secondary hypotheses (supportive outcomes):

In addition to the primary and key secondary outcomes, we will assess the impact of SH+ on three secondary domains, each addressed by the following hypotheses:

- H3a—Maternal mental health and functioning: SH+ will reduce maternal depression, anxiety, general stress, posttraumatic stress symptoms, and functional impairment at 12 months, relative to EUC.
- H3b—Parenting practices: SH+ will enhance positive parenting practices and reduce harsh discipline at 12 months, relative to EUC.
- H3c—Child wellbeing and functioning: SH+ will reduce children’s emotional and behavioral problems and improve their self-reported psychosocial wellbeing at 12 months, relative to EUC.

A detailed description of the primary, key secondary, secondary, and exploratory outcomes is provided in the “Outcomes {12}” section, below.

### Trial design {8}

This is a two‑arm, parallel‑group, superiority cluster‑randomized controlled trial (cRCT) with a 1:1 allocation of 24 villages (clusters) to (i) SH+ plus Enhanced Usual Care or (ii) Enhanced Usual Care only. The trial is designed to test the superiority of SH+ in reducing maternal psychological distress at 12 months post-intervention (primary outcome) and to evaluate its effects on parent-reported child psychosocial wellbeing at 12 months as a key secondary outcome, tested hierarchically following the primary outcome.

Cluster randomization at the village level is employed because SH+ is delivered in group sessions within the community and to minimize contamination that could occur if mothers in the same village were assigned to different study arms.

## Methods: participants, interventions, and outcomes

### Study setting {9}

The trial will take place in Rhino Refugee Settlement, a protracted displacement setting in Uganda’s West Nile sub‑region covering roughly 225 km^2^ across Terego and Madi‑Okollo districts. Established in the 1960 s and expanded dramatically after renewed conflict in South Sudan (2013–2016), it now hosts approximately 170,000 registered refugees as of 31 January 2025, over 90% of whom are South Sudanese—mostly women and children [19]. The settlement consists of more than 80 villages governed by elected Refugee Welfare Councils and organized into six zones (Eden, Ocea, Odobu, Siripi, Tika, and Omugo/Ofua) [20].

Under Uganda’s self-reliance policy, households receive small plots of land averaging 0.6 acres for shelter and subsistence farming [21]. However, primary health‑care posts operate with chronic staff shortages and frequent drug stock‑outs [14, 22]. Formal mental‑health services are extremely limited, consisting of a single visiting psychiatric officer and intermittent NGO-based counseling teams [22, 23]. “Overthinking” (*tamaku tafkir*) is a locally salient idiom of distress [14, 22], and protection risks such as sexual and gender‑based violence and inter-ethnic tensions remain high [22, 24].

All study activities—including recruitment, intervention delivery, and data collection—will be conducted within the selected villages of the refugee settlement, in collaboration with local authorities and community leaders.

### Eligibility criteria {10}

The trial will be conducted in 24 villages (clusters) within Rhino Camp Refugee Settlement. To identify potential villages, we will collaborate with local government offices, the Office of the Prime Minister (OPM), and Refugee Welfare Councils. To be eligible for inclusion, villages must have a population of at least 1000 residents and be accessible by motorized transport. Villages will be excluded if they have high levels of urbanization or a prior SH+ participation rate above 5% to prevent contamination. If selected villages are too small to meet per-cluster sample size targets, they may be combined with neighboring villages to form larger units prior to randomization.

Within each selected village, refugee mothers are eligible to participate if they are aged 18 years or older; are the mother of a child aged 3–4 years at enrollment; can speak and understand Juba Arabic; and do not plan to move from the settlement within the next 12 months. A further inclusion criterion is the presence of moderate psychological distress, defined as a score of 5 or higher on the Kessler-6 (K6) psychological distress scale [17].

Exclusion criteria for mothers include the following: current participation in another mental health intervention; having received SH+ in the past or being familiar with it; or imminent risk of suicide, as assessed by the suicidality subscale of the Mini International Neuropsychiatric Interview [25]. Mothers will also be excluded for observable signs of psychosis, manic behaviors, or intellectual disability that would impede participation, as determined by trained research assistants.

For children to be included, they must be between 3 and 4 years old at the time of enrollment and reside with their participating mother. Children will be excluded if they have a known cognitive impairment or developmental delay that would preclude participation in age-appropriate assessments. This is based on maternal report and a case-based assessment by trained enumerators, as formal diagnostic assessments are not feasible in this setting. If a mother has multiple eligible children, the index child will be the one with the most recent birthday.

SH+ facilitators will be recruited from the refugee and host communities. They must have completed at least a secondary education, be proficient in Juba Arabic and English, and have prior experience working in the settlement.

### Who will take informed consent? {26a}

Trained local enumerators proficient in Juba Arabic and English will conduct the informed consent process with potential participants in their preferred language. All enumerators will undergo a comprehensive training program covering the study protocol, ethical principles of research with vulnerable populations, and procedures for obtaining and documenting informed consent.

The consent process will be conducted in a private and confidential setting. The enumerator will provide a detailed verbal explanation of the study, including the study purpose, procedures, duration; potential risks and benefits; the voluntary nature of participation; and the right to withdraw at any time without consequence. This information will also be provided in a written information sheet.

For literate participants, written informed consent will be documented by a signature on the consent form. For participants who are illiterate, the information sheet will be read and explained by the enumerator in the presence of an impartial witness (who is not part of the study team). If the participant consents, she will provide a thumbprint, and the witness will sign to confirm that the information was accurately explained and that consent was freely given.

For child participation, mothers will provide written informed consent on behalf of their child. Additionally, a simplified oral assent process will be conducted with each child to ensure they are willing to participate in the assessments.

### Additional consent provisions for collection and use of participant data and biological specimens {26b}

This trial does not involve the collection of biological specimens. Consent for the use of anonymized participant data in future secondary analyses will be sought as part of the main informed consent process.

## Interventions

### Explanation for the choice of comparators {6b}

The control group will receive Enhanced Usual Care (EUC), consistent with Tol et al.’s trial in this setting [14]. A no-treatment control condition was deemed inappropriate given the high levels of psychological distress in this population. EUC was therefore selected as an ethical active comparator, providing all participants with psychoeducation, information on existing mental health services, and referrals to care. This approach ensures all participants receive a baseline level of support, controls for attention and common factors, and mitigates potential nocebo effects associated with a no-treatment control.

The intervention group will receive SH+ in addition to EUC. This design directly tests the added value of SH+ over an active control. By providing EUC in both arms, we more precisely estimate the effects attributable to SH+. The choice of SH+ is based on prior evidence demonstrating its safety and effectiveness in reducing psychological distress in this specific refugee population [14], its design for scalability in low-resource settings, and its potential to influence the theorized mechanisms of change, including maternal wellbeing and decision-making.

### Intervention description {11a}

Participants in both arms of the trial will receive Enhanced Usual Care (EUC). Participants allocated to the intervention arm will receive Self-Help Plus (SH+) in addition to EUC.

#### Enhanced Usual Care (EUC)

All enrolled participants (mothers) will receive EUC, which consists of a single 15-min psychoeducation session delivered individually by a trained research assistant under the supervision of a clinical psychologist from our implementation partner, *vivo international*. The session is guided by a predetermined script, focusing on the locally salient concept of “overthinking” [22] and introducing brief, practical self-management techniques. Following the session, participants are provided with standardized information about available mental health resources within Rhino Refugee Settlement, including services provided by UNHCR partner organizations and a network of community-based health workers trained to offer basic psychosocial support. EUC is delivered to all participants after the baseline assessment; for those in the intervention arm, it precedes the start of SH+.

#### Self-Help Plus (SH+)

Participants in the intervention arm will additionally receive Self-Help Plus (SH+), a World Health Organization (WHO) brief, low-intensity intervention designed for stress management in communities affected by adversity [12, 13]. SH+ is based on Acceptance and Commitment Therapy (ACT) and is delivered in a group-based format (up to 30 participants) over five weekly sessions, each lasting approximately 2 h.

The core content is delivered via pre-recorded audio material (using the Juba Arabic adaptation), which is integrated with individual exercises and guided group discussions. Participants also receive an illustrated, low-literacy self-help book to review strategies and practice skills between sessions. This multimedia format, guided by a facilitator manual, is designed to enhance fidelity and minimize extensive training demands, making it suitable for scalable delivery in low-resource settings.

Sessions are co-facilitated by pairs of trained local lay-providers who reside in the settlement, have completed at least secondary education, and are proficient in both Juba Arabic and English. The training and supervision of these facilitators are led by experienced mental health specialists from *vivo international Uganda*. These specialists first completed a comprehensive Training of Trainers (ToT) conducted by the research team using WHO materials and guidelines. The ToT equipped them to deliver the SH+ training, conduct fidelity assessments, provide supervision, and manage referral pathways. Following this, the *vivo* specialists provide the facilitators with 5 days of specialized SH+ training and a 3-day supervised, hands-on pilot with test participants. Ongoing weekly supervision from these specialists includes a review of session checklists, feedback on delivery, and guidance on managing group dynamics to ensure fidelity to the SH+ model.

SH+ materials have been professionally translated and culturally adapted into Juba Arabic following established best practices [26], and are publicly available on the WHO website [27].

### Criteria for discontinuing or modifying allocated interventions {11b}

Participants are free to withdraw from the study interventions at any time upon their request without providing a reason. The intervention facilitators or the clinical supervisor may also recommend withdrawal for a participant if they determine that continued participation is not in the participant’s best interest. Withdrawal will not affect access to any services or benefits available in the settlement.

Specific criteria for discontinuation from the intervention include the identification of imminent risk of suicide or harm to self/others, or the emergence of severe, acute psychiatric symptoms (e.g., psychosis or manic episodes) that require immediate, specialized care beyond the scope of this intervention. In such cases, the participant will be immediately referred to the appropriate mental health services within the settlement, and their participation in the intervention will be discontinued. Discontinuation from the intervention does not imply withdrawal from the study; where safe and consented, participants will be followed for outcome assessments in line with the intention-to-treat approach.

This protocol does not specify conditions for modifying the allocated interventions, as both the EUC and SH+ interventions are standardized and delivered according to a manualized protocol to ensure fidelity. No therapeutic content modifications are permitted; facilitators may make brief, culturally appropriate clarifications to enhance comprehension without altering core content or procedures. All instances of participant withdrawal or discontinuation from the intervention will be documented and any safety-related referrals will be reported in accordance with the trial’s adverse-event procedures.

### Strategies to improve adherence to interventions {11c}

To ensure the interventions are delivered as intended, all facilitators will complete a comprehensive, standardized training program based on the WHO SH+ manual, including role-plays and competency assessments. Fidelity will be further promoted through the use of structured materials, including pre-recorded audio content and facilitator manuals for SH+ and a detailed script for EUC. SH+ facilitators will also participate in weekly supervision meetings with an experienced clinical supervisor to discuss challenges and reinforce adherence to the protocol. Finally, a clinical supervisor will directly observe a random sample of at least 10% of all SH+ sessions using a standardized fidelity checklist, and facilitators will complete their own checklists after each session to document activities and note any deviations and corrective actions.

### Relevant concomitant care permitted or prohibited during the trial {11d}

Participants will be permitted to access and continue all forms of usual care available in the Rhino refugee settlement throughout the trial period. This includes services provided by government health facilities, NGOs, and other humanitarian organizations. No specific treatments or therapies are prohibited; participants may initiate, continue, or modify usual care, including emergency/acute care and pharmacotherapy, at any time.

To monitor for potential confounding, information regarding participants’ use of other psychosocial or mental health support services will be collected at all follow-up assessment points. Participants who are concurrently enrolled in another formal psychosocial intervention trial will be excluded at screening; if such enrollment occurs after randomization, it will not constitute a protocol deviation but will be documented as concomitant care and addressed analytically.

### Provisions for post-trial care {30}

Given the low-intensity and non-invasive nature of the interventions, no specific post-trial clinical care is required. All participants, regardless of trial arm, will continue to have access to the standard health and psychosocial services available in Rhino Camp Refugee Settlement after the study concludes. These include primary health-care services provided by the Ministry of Health, UNHCR, and partner NGOs.

In cases where participants experience distress or mental health deterioration during or after the trial, they will be referred to existing mental health service providers within the settlement, including the psychiatric officer at the health center and NGO counseling teams. Contact details for these referral options are provided to all participants during the informed consent process.

Upon completion of the trial, community debriefing meetings will be organized in collaboration with settlement leadership, the Office of the Prime Minister, and implementing partners to communicate results and provide information about ongoing psychosocial support initiatives. This approach helps to ensure that participants and their communities remain connected to available services and are informed about future intervention opportunities.

### Outcomes {12}

The outcomes of this trial are designed to capture both the direct effects of Self-Help Plus (SH+) on maternal mental health and potential intergenerational effects on preschool-aged children. Guided by the study’s conceptual model, outcomes are organized into four categories: (i) the primary outcome, capturing maternal psychological distress; (ii) the key secondary outcome, capturing parent-reported child psychosocial wellbeing; (iii) secondary outcomes, capturing broader maternal mental health and functioning, parenting practices, and additional child psychosocial outcomes; and (iv) exploratory outcomes, capturing ancillary domains that will be analyzed and reported in separate planned publications. Primary, key secondary, and secondary outcomes will be collected at baseline (T0), 3 months post-intervention (T1), and 12 months post-intervention (T2). At baseline, sociodemographic and other potential confounding variables will be collected to describe the sample, a subset of which will be controlled for in statistical analyses (see the “Statistical methods for primary and secondary outcomes {20a}” section); these variables are detailed in Supplementary Table S1. The 12-month follow-up (T2) serves as the primary endpoint for the primary and key secondary hypotheses; outcomes measured at T1 will be analyzed as supportive time points to characterize trajectories. All primary, key secondary, and secondary outcomes will be analyzed as continuous total scores. Prespecified exploratory outcomes (including socio-behavioral skills, cognitive development, and other ancillary domains) are not part of the confirmatory testing strategy and will be reported in separate planned publications (see Supplementary Table S2).

#### Primary outcome

The primary outcome is maternal psychological distress assessed at 12 months post-intervention (T2), measured using the Kessler-6 (K6) [17], a 6-item scale assessing non-specific symptoms of depression and anxiety over the past 30 days. Each item is scored on a 0–4 Likert scale (0 = “none of the time” to 4 = “all of the time”), with total scores ranging from 0 to 24; higher scores indicate greater distress.

#### Key secondary outcome

The key secondary outcome is parent-reported child psychosocial wellbeing assessed at 12 months post-intervention (T2) using the Kiddy-KINDL^R^ (parent-report version) [18], a 24-item instrument assessing emotional wellbeing, self-esteem, family and peer relationships, and daily functioning. Consistent with KINDL scoring conventions, the total health-related quality-of-life score is computed from the 20 non-school items ranging from 1 (“never”) to 5 (“all of the time”), and transformed to a 0–100 scale, where higher scores indicate greater wellbeing.

Both instruments have demonstrated good reliability in prior studies and were linguistically adapted for use in Juba Arabic through standardized forward–backward translation.

#### Secondary outcomes

##### Maternal mental health and functioning

Depression: Assessed with the 9-item Patient Health Questionnaire (PHQ-9) [28]. Each item is scored on a 4-point Likert scale from 0 (“not at all”) to 3 (“nearly every day”), with total scores ranging from 0 to 27. Higher scores indicate more severe depressive symptoms.

Anxiety: Assessed with the 7-item Generalized Anxiety Disorder scale (GAD-7) [29]. Each item is scored on a 4-point Likert scale from 0 (“not at all”) to 3 (“nearly every day”), with total scores ranging from 0 to 21. Higher scores indicate greater anxiety symptom severity.

Post-traumatic stress symptoms: Measured using the 6-item PTSD Checklist–Civilian Version (PCL-C) [30]. Each item is rated on a 5-point scale from 1 (“not at all”) to 5 (“extremely”), yielding a total score between 6 and 30. Higher scores reflect greater severity of post-traumatic stress symptoms.

Perceived stress: Assessed using the 4-item Perceived Stress Scale (PSS-4) [31]. Items are scored on a 5-point scale from 0 (“never”) to 4 (“very often”), with two items reverse-coded. Total scores range from 0 to 16, with higher scores indicating greater perceived stress.

Subjective wellbeing: Measured with the 5-item WHO Well-Being Index (WHO-5) [32]. Each item is rated on a 6-point scale from 0 (“at no time”) to 5 (“all of the time”). The raw total score is multiplied by 4 to yield a final standardized score from 0 to 100, with higher scores representing greater psychological wellbeing.

Functional impairment: Assessed using the 12-item WHO Disability Assessment Schedule 2.0 (WHODAS 2.0) [33]. Items assess difficulties in six domains of functioning, each rated from 0 (“none”) to 4 (“extreme or cannot do”). Higher total scores, ranging from 0 to 48, indicate greater functional impairment.

Psychological flexibility: Assessed with the 7-item Acceptance and Action Questionnaire-II (AAQ-II) [34]. Items are scored on a 7-point Likert scale from 1 (“never true”) to 7 (“always true”) and reverse-coded so that higher total scores (ranging from 7 to 49) indicate greater psychological flexibility.

##### Child wellbeing and functioning

Emotional and behavioral problems: Measured using the parent-reported 17-item Pediatric Symptom Checklist (PSC-17) [35]. Items are rated on a 3-point scale (0 = “never a problem”, 1 = “sometimes a problem”, 2 = “often a problem”), with total scores ranging from 0 to 34. Higher scores indicate greater emotional and behavioral difficulties.

Self-reported psychosocial wellbeing: Assessed with the 12-item child-report version of the Kiddy-KINDL^R^ [36]. Each item is rated on a 3-point Likert scale from 1 (“never”) to 3 (“very often”), with total scores ranging from 12 to 36. Higher scores reflect greater psychosocial wellbeing as perceived by the child.

##### Parenting practices

Positive parenting: Measured using the 6-item Positive Parenting Subscale of the Alabama Parenting Questionnaire (APQ) [37]. Each item is rated on a 5-point scale from 1 (“never”) to 5 (“always”). Higher total scores, ranging from 6 to 30, indicate more frequent engagement in positive parenting behaviors.

Disciplinary practices: Measured with the 11-item Discipline Module from the UNICEF Multiple Indicator Cluster Surveys (MICS6) [38]. This module consists of 11 questions about the frequency of violent and non-violent disciplinary actions taken by caregivers in the past month. Items are rated from 0 (“never”) to 3 (“three or more times”), producing a total score between 0 and 33. Higher scores indicate greater overall use of disciplinary methods.

**Fig. 1:**
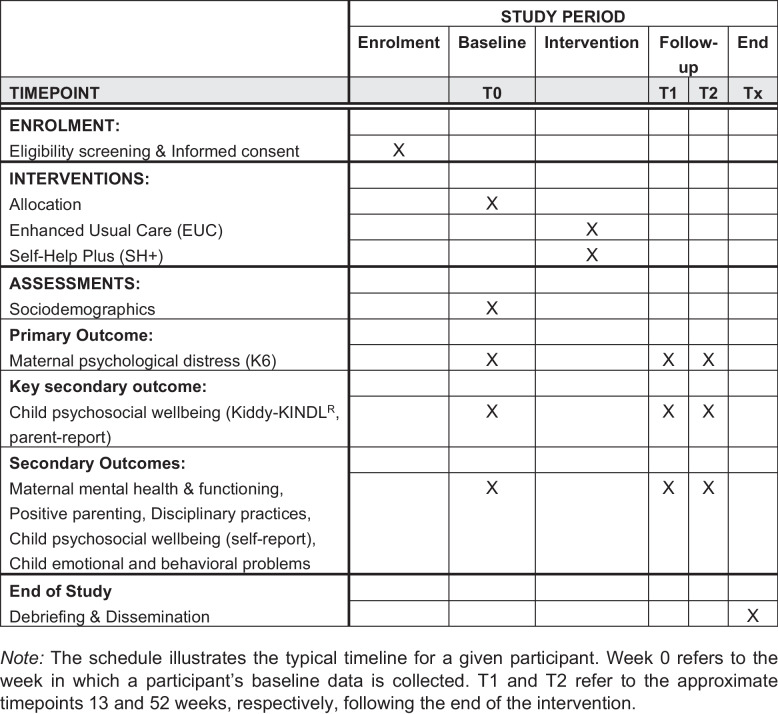
SPIRIT schedule of enrollment, interventions, and assessments for the SEED trial

Table 1 provides a summary of the primary, key secondary, and secondary outcome measures, and Fig. 1 presents the schematic timeline of enrollment, interventions, and assessments.

**Table 1:** Primary, key secondary, and secondary outcomes for the SEED trial

Outcome	Instrument	Follow-up	
		**T1**	**T2**
**Primary outcome:**			
Maternal psychological distress	Kessler Psychological Distress Scale (K6) [17]	X	X
**Key secondary outcome:**			
Child psychosocial wellbeing (parent-report)	Kiddy-KINDL^R^ - Parent Report [18]	X	X
**Secondary outcomes:**			
**Maternal mental health**			
Depression	Patient Health Questionnaire-9 (PHQ-9) [39]	X	X
Anxiety	Generalized Anxiety Disorder 7-item scale (GAD-7) [29]	X	X
Post-traumatic stress	Post-Traumatic Checklist 6-item Civilian Version (PCL-C) [30]	X	X
Perceived stress	Perceived Stress Scale 4-item version (PSS-4) [31]	X	X
**Maternal wellbeing and functioning**			
Subjective wellbeing	WHO-5 Well-Being Index [32]	X	X
Functional impairment	15-item version of the World Health Organization Disability Assessment Schedule 2.0 (WHODAS-II) [40]	X	X
Psychological flexibility	Acceptance and Action Questionnaire - version 2 (AAQ-2) [34]	X	X
**Child wellbeing and functioning**			
Emotional and behavioral problems	Pediatric Symptom Checklist (PSC-17) [35]	X	X
Psychosocial wellbeing (self-report)	Kiddy-KINDL^R^ – self-report [36]	X	X
**Parenting practices**			
Positive parenting	6-item Positive Parenting Subscale of the Alabama Parenting Questionnaire (APQ) [37]	X	X
Disciplinary practices	11-Item Discipline Module of the Multiple Indicator Cluster Surveys (MICS) [38]	X	X

#### Exploratory outcomes

To provide a richer understanding of the intervention’s impact, data on several exploratory and contextual variables will be collected. These include measures of food security (Food Insecurity Experience Scale (FIES)) [41, 42], household dietary diversity (HDDS) [43], child malnutrition (height-for-age *z*-score (HAZ)) [44, 45], and social capital (group membership, participation, and trust). These will be assessed at all three timepoints (T0, T1, T2), except HAZ, which will be measured only at T0 and T2. In addition, socio-behavioral skills will be assessed at baseline (T0) and 12 months (T2) using incentivized tasks: patience is measured with an investment task adapted from Andreoni and Sprenger [46]; risk tolerance with a bomb risk-elicitation task adapted from Crosetto and Filippin [47]; and prosociality with four binary allocation games adapted from Fehr and colleagues and Bauer and colleagues [48, 49]. We will also assess child cognitive skills, including mathematics ability (free counting, number comparison, Give-N, addition/subtraction tasks) [50], language ability (TIFALDI) [51], spatial ability (TOSA) [50], and theory-of-mind (“surprise outcome” and “surprise content” tasks) [52, 53] at baseline (T0) and the 12-month follow-up (T2). All exploratory instruments and tasks will be culturally adapted and pilot-tested during a 5-day training in Arua with Juba Arabic–speaking research assistants; enumerators receive supervised practice (including role-plays and field piloting with mother–child dyads) to standardize administration before data collection. Supplementary Table S2 provides an overview and description of exploratory outcomes and measures. These exploratory outcomes are not part of the confirmatory testing strategy and will be reported in separate, dedicated analyses.

### Participant timeline {13}

**Fig. 2:**
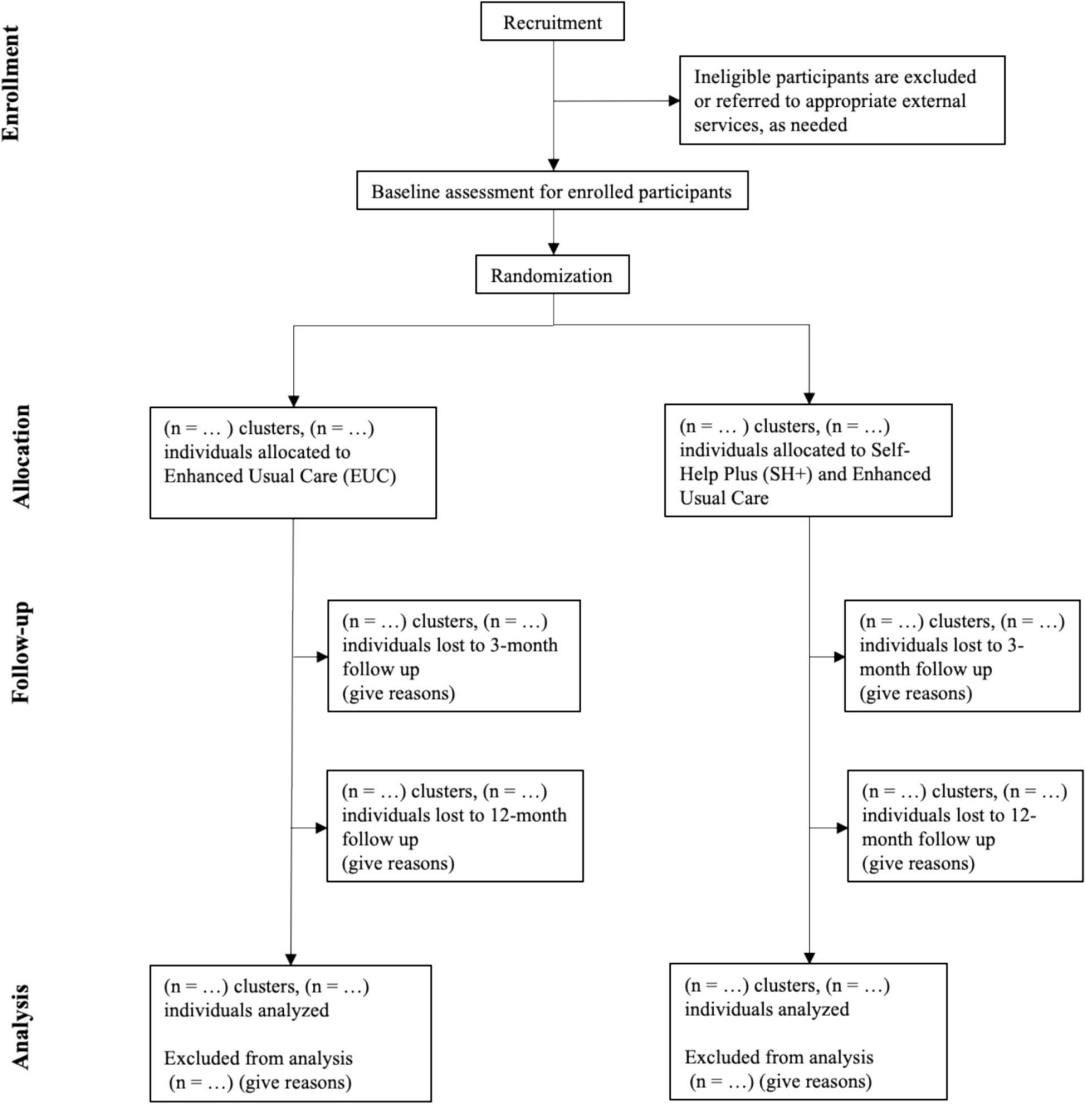
SPIRIT flow diagram of the phases of the SEED trial

The SPIRIT participant flow diagram is shown in Fig. 2. Baseline data will be collected immediately after participants complete screening, provide informed consent, and are enrolled in the study. All participants will then receive a single session of Enhanced Usual Care (EUC) before randomization. Those assigned to the intervention arm will then participate in the Self-Help Plus (SH+) program, which consists of five weekly group sessions. Follow-up assessments will be conducted at 3 and 12 months post-intervention.

### Sample size {14}

We determined the required sample size using *PowerUp!* [54], focusing on the primary outcome of maternal psychological distress (Kessler-6) [17] at the 12-month follow-up. Calculations were based on a two-arm cluster-randomized design with equal allocation and equal expected cluster sizes.

Our objective is to achieve 90% statistical power to detect a minimal clinically important difference (MCID), defined as Cohen’s *d* = 0.20, for the primary outcome using a two-tailed significance level (*α*) of 0.05. The parent-reported Kiddy-KINDL^R^ at T2 is specified as a key secondary outcome and will be tested using a hierarchical fixed-sequence procedure: K6 will be tested first at *α* = 0.05, and Kiddy-KINDL^R^ will be formally tested at *α* = 0.05 only if the primary test is statistically significant. This approach controls the overall Type I error rate across the primary and key secondary outcomes without requiring alpha adjustment of the individual tests [55]. While modest, an effect size of this magnitude (*d* = 0.20) is meaningful for a low-intensity, scalable intervention delivered by lay facilitators. In high-burden humanitarian settings, even small average improvements in psychological wellbeing can translate into substantial population-level benefits and policy relevance [56].

For the power calculation, we used an intra-cluster correlation coefficient (ICC) of 0.012, based on the values observed in the earlier SH+ cluster-RCT in the same setting and population [14]. The SH+ intervention is designed for delivery in groups of up to 30 individuals. To account for potential attrition over the 12-month study period, we conservatively assume a 20% dropout rate. Consequently, we plan to recruit 30 mother–child dyads per cluster, with an expected 24 analyzed per cluster at endline. Based on these parameters (power = 0.90, *α* = 0.05, *d* = 0.20, ICC = 0.012, *m* = 24 analyzed per cluster), *PowerUp!* indicates that 24 clusters (12 per arm) are required. This yields a total target sample of 720 mother–child dyads across 24 villages within Rhino Refugee Settlement.

These calculations are conservative, assuming no variance reduction from baseline adjustment. Because the primary analyses will control for baseline outcome values and cluster-level covariates, realized statistical power is expected to be at least as high as the nominal estimate. Secondary outcomes are not used to determine trial success and are not specifically powered; however, under the same design parameters, this sample size provides reasonable power to detect small-to-moderate effects for continuous secondary outcomes.

We will document actual cluster sizes, attrition, and observed ICCs during implementation and report effect estimates with 95% confidence intervals to reflect the precision achieved.

### Recruitment {15}

Access to the settlement will be obtained through formal approval from the Office of the Prime Minister (OPM). Following approval, the research team will engage camp authorities to present the study and receive operational feedback. As part of this process, camp authorities will provide updated lists of villages and population estimates, which—together with our predefined village inclusion criteria (see the “Eligibility criteria {10}” section)—will be used to compile the frame of eligible villages. To facilitate recruitment in smaller villages, contiguous villages may be combined to form a single study cluster prior to randomization. An independent researcher not involved in the study will then create a computer-generated randomized list of eligible villages. Our field coordinator will approach village leaders sequentially in that randomized order to invite their village to participate. This process will proceed until 24 clusters (villages or pre-combined units) are included (12 per arm).

We will then convene an inception meeting with village leaders from each of the 24 included clusters, Refugee Welfare Council (RWC) leadership, and OPM representatives to present the trial, discuss implementation logistics, and incorporate feedback. All communications emphasize the voluntary nature of participation, participants’ right to withdraw, and that access to usual services is unaffected by participation or allocation.

Within each selected cluster, we will work in close collaboration with the local RWC representative to prepare a comprehensive roster of residents who meet the study’s individual inclusion criteria. Each roster will be randomized before fieldwork; enumerators will approach prospective participants sequentially in that randomized order, confirm eligibility in a private setting, and obtain informed consent. Recruitment will proceed until 30 mother–child dyads are enrolled per cluster. Households that are ineligible, decline, or cannot be reached will be recorded before proceeding to the next entry.

Assessments will be conducted at the most suitable nearby location to balance privacy, accessibility, and efficiency. Where feasible, this will be within the village; in other cases—particularly for combined clusters—assessments will occur at a nearby central venue serving multiple villages.

Recruitment teams will maintain standardized logs (households approached, screened, consented, enrolled; reasons for ineligibility/refusal; assessment dates and venues) to support CONSORT-compliant reporting and real-time monitoring of accrual against targets. Coordination with camp authorities, village leaders, and RWCs will continue throughout implementation to address local scheduling constraints and logistical issues, while at the same time maintaining adherence to the study protocol and all ethical safeguards.

## Assignment of interventions: allocation

### Sequence generation {16a}

Randomization will occur at the cluster (village or pre-combined village unit) level with allocation performed at a 1:1 ratio to one of two arms: (i) Self-Help Plus (SH+) with Enhanced Usual Care (EUC), or (ii) EUC only. The allocation sequence will be implemented using a sequentially numbered, opaque, sealed envelopes (SNOSE) procedure [57]. An equal number of cards indicating each of the two trial arms will be prepared. These cards will be folded and placed into identical, opaque envelopes, which are then sealed and shuffled. This preparation will be conducted by a collaborator of the local research team who is uninvolved in the recruitment process.

### Concealment mechanism {16b}

Allocation concealment is ensured using the SNOSE method. Opaque, sequentially numbered envelopes will keep the allocation concealed until the moment they are opened, and they will be sealed to make any tampering evident. Because envelopes are indistinguishable and unopened prior to the draw, the allocation is unknown to field staff, RWC collaborators, and participants up to the moment of assignment at the public allocation meeting with village representatives.

### Implementation {16c}

A senior member of the local research team will oversee the allocation process. The allocation itself will be implemented during a central meeting attended by representatives from all 24 participating villages. At this meeting, the procedure will be explained, after which each village representative will publicly draw one sealed envelope at random to determine their cluster’s assignment. This participatory method ensures transparency and community understanding of the randomization process. Once a cluster is allocated, all recruited participants from that cluster are assigned to the corresponding trial arm. Participant enrollment and baseline data collection are conducted prior to cluster allocation.

## Assignment of interventions: blinding

### Who will be blinded {17a}

Because this is a cluster-randomized community trial, blinding of participants and facilitators is not feasible: participants will necessarily be aware of whether they are attending Self-Help Plus (SH+) sessions or receiving Enhanced Usual Care (EUC) only. However, several safeguards are in place to minimize bias in data collection and analysis.

All enumerators, field supervisors, and data entry personnel are blinded to allocation. Enumerators who conduct baseline, 3-month, and 12-month assessments will not be informed of cluster assignment and will have no involvement in intervention delivery.

To prevent unblinding during fieldwork, all intervention activities (SH+ sessions, facilitator meetings, and supervision) are managed by a separate implementation team. This team operates independently of the research staff responsible for screening and outcome measurement. Study databases use coded cluster identifiers, and a master linkage file connecting codes to allocation status is accessible only to the principal investigator and data manager.

### Procedure for unblinding if needed {17b}

Given the low-risk nature of the interventions, we do not foresee any circumstances where unblinding would be required.

## Data collection and management

### Plans for assessment and collection of outcomes {18a}

All data collection will be conducted by trained, Juba Arabic–speaking enumerators who are independent of the SH+ facilitation team. Enumerators will undergo a 5-day intensive training covering ethical procedures, standardized interviewing techniques, task administration, and child engagement strategies. Training will include extensive role-playing, piloting with test participants, and feedback sessions to ensure comprehension and reliability.

Baseline (T0), 3-month (T1), and 12-month (T2) assessments will be conducted at pre-determined venues nearby (see the “Recruitment {15}” section). Enumerators will use tablet-based questionnaires programmed in KoBoToolbox, which includes built-in range checks, skip patterns, and validation rules to minimize data-entry errors. Standard operating procedures (SOPs) will guide all aspects of data collection, including participant identification, consent documentation, and interview termination criteria.

To ensure the cultural validity and clarity of all measures, tools will be translated into Juba Arabic, then refined through piloting with test participants during enumerator training. Feedback from field staff and test participants will inform minor linguistic or contextual adjustments (e.g., colloquial expressions, culturally appropriate child task materials). The full data collection forms are available from the corresponding author upon reasonable request.

Supervisors will conduct random spot checks and daily data reviews to ensure adherence to protocols and assess data completeness. Any inconsistencies will be flagged, reviewed with enumerators, and corrected on the same day. Supervisors will also document field conditions, interruptions, or participant distress events to contextualize data quality.

### Plans to promote participant retention and complete follow-up {18b}

During screening, we exclude individuals who report plans to relocate within the 12-month study period. Participant retention will be supported by regular communication through RWCs and village leaders, flexible scheduling to accommodate livelihood activities, and provision of light refreshments and safe play spaces for children during assessments. To minimize participant burden, the brief 3-month follow-up assessment will be conducted at participants’ homes. At enrollment, we collect multiple contact details for each participant for tracking purposes, including details for alternate contacts who can help locate the participant if temporarily away. Between assessments, we will conduct three brief administrative check-in calls at set intervals to confirm and update contact information, verify anticipated absences, and refresh alternate contact details. Participants who relocate within Rhino Refugee Settlement will be traced via their RWC representative. If a participant moves outside the settlement, where feasible, we will conduct assessments remotely to ensure a more complete dataset for intention-to-treat analyses.

### Data management {19}

Data will be collected and managed using a secure, GDPR-compliant electronic data capture system. Each participant will be assigned a unique study ID; personally identifying information will be stored separately from survey responses. Trained enumerators will enter data directly onto password-protected tablets using the KoboCollect application. This method minimizes data entry errors by enabling real-time validation checks (e.g., for data types and ranges) during data collection.

Data will be uploaded daily from the tablets to a secure, password-protected server hosted by KoboToolbox. To further ensure data quality, the core research team based at Uppsala University will perform daily checks on uploaded records for completeness and identify any inconsistencies or potential errors for immediate follow-up with the field team.

All electronic data will be encrypted both during transmission and while stored. To prevent data loss, regular backups of the dataset will be maintained on a secure, access-controlled server at Uppsala University.

Following trial completion, the cleaned and de-identified dataset will be archived in accordance with Uppsala University’s data governance policies and FAIR data principles. Access to the anonymized dataset for secondary analysis will be granted upon reasonable request and approval by the principal investigator and the relevant ethics committees.

### Confidentiality {27}

All research staff, including enumerators, facilitators, and supervisors, will receive comprehensive training on the importance of maintaining participant confidentiality and data security protocols.

Participant information will be protected at every stage of the study. All electronic data collected via tablets will be password protected and encrypted. All participants will be assigned a unique, non-identifying participant ID number upon enrollment. This ID number will be used on all data collection forms and in the electronic dataset to ensure anonymity.

The file linking participant names to their ID numbers will be stored separately from the main research data. This linking file will be accessible only to a limited number of authorized senior research staff for necessary follow-up and data management purposes.

Consent forms linking names to ID codes will be stored separately in locked filing cabinets at the field office in Uganda, accessible only by the field coordinator.

In all publications and presentations resulting from this research, data will be presented in an aggregated and anonymized format, ensuring that no individual participants can be identified.

### Plans for collection, laboratory evaluation and storage of biological specimens for genetic or molecular analysis in this trial/future use {33}

Not applicable. No biological specimens will be collected, and no genetic or molecular analyses will be conducted in this trial.

## Statistical methods

### Statistical methods for primary and secondary outcomes {20a}

The primary analysis will follow the intention-to-treat (ITT) principle, including all randomized participants in their assigned groups regardless of intervention adherence. The primary outcome is maternal psychological distress (Kessler-6) at 12 months post-intervention (T2). The key secondary outcome is parent-reported child psychosocial wellbeing (Kiddy-KINDL^R^ Parent Report) at T2. To control the overall type I error rate across the primary and key secondary outcomes, we will use a hierarchical fixed-sequence testing procedure: K6 will be tested first at two-sided *α* = 0.05, and Kiddy-KINDLR will be formally tested at two-sided *α* = 0.05 only if the primary test is statistically significant [55]. For the primary and secondary outcomes, we will use linear mixed-effects models (LMM) with random intercepts for villages and participants. The primary estimand for hypothesis testing is the T2 outcome value (final value), with adjustment for the baseline (T0) value of the same outcome when measured at baseline. A pre-specified covariate-adjusted model will be run as a robustness check, additionally adjusting for maternal age and education, child age and sex, household size, and time in the settlement. Results will be presented as adjusted mean differences with 95% confidence intervals and as standardized mean differences (Hedges’ *g*). Outcomes measured at 3 months (T1) will be analyzed analogously as supportive time points to characterize trajectories.

### Interim analyses {21b}

No interim analyses for efficacy or futility are planned.

### Methods for additional analyses (e.g., subgroup analyses) {20b}

Exploratory subgroup analyses will be conducted to assess whether treatment effects vary by baseline maternal psychological distress, trauma exposure, and parenting practices. Effect moderation will be examined by including interaction terms (intervention × moderator) within the LMM framework.

### Methods in analysis to handle protocol non-adherence and any statistical methods to handle missing data {20c}

A per-protocol analysis will be conducted as a sensitivity analysis, including only participants who demonstrate adequate adherence to the intervention protocol. Missing data will be handled using maximum-likelihood estimation within the mixed-effects models, which is valid under the missing-at-random (MAR) assumption. If > 10% of observations are missing at T2, we will perform multiple imputation as a sensitivity analysis.

### Plans to give access to the full protocol, participant-level data and statistical code {31c}

Upon publication of primary results, we will make the final study protocol, anonymized participant-level dataset, and statistical code used for the analysis publicly available on the Open Science Framework (OSF).

[https://osf.io/46qhb/?view_only=82598d792f304ee692fd636c94d5e7eb].

## Oversight and monitoring

### Composition of the coordinating center and trial steering committee {5d}

The trial will be coordinated from Uppsala University, Sweden, which will handle overall study management, including ensuring ethics compliance, data oversight, and reporting. The Trial Steering Committee (TSC), comprising all co-authors, will supervise the study, convening regularly to monitor progress and resolve emerging issues.

### Composition of the data monitoring committee, its role and reporting structure {21a}

Due to the low-risk nature of the interventions and their prior use in similar settings without evidence of serious harm, a separate data monitoring committee will not be established. Oversight of trial conduct and participant safety will instead be provided by the Trial Steering Committee and the relevant ethics committees.

### Adverse event reporting and harms {22}

SH+ is a brief, low-intensity stress-management intervention and prior studies have not indicated intervention-related harms as a common concern. Nonetheless, clinically significant deterioration or safeguarding concerns may be identified during sessions or assessments. Adverse events will be captured non-systematically through spontaneous reporting and facilitator/staff observation. Any adverse event will be reported to the Trial Steering Committee within 24 h. The Trial Steering Committee will evaluate severity and assess whether the event is related to the study intervention. Serious adverse events will be reported to the relevant ethics committees for review. In trial reporting, adverse events identified through spontaneous reporting will be summarized descriptively by trial arm, consistent with CONSORT-SPI 2018 [58] and guided by CONSORT Harms 2022 [59] as appropriate.

### Frequency and plans for auditing trial conduct {23}

The trial may be audited at any time by the Kabale University Research Ethics Committee (KAB-REC) to ensure compliance with the approved protocol. The timing of these audits is at the discretion of KAB-REC and will be independent of the investigators and the sponsor.

### Plans for communicating important protocol amendments to relevant parties (e.g., trial participants, ethical committees) {25}

Any significant changes to the protocol such as amendments to eligibility criteria, outcomes, or analysis plans will be communicated to all relevant stakeholders, including investigators, ethics committees, trial registries, journals, participants, and regulatory authorities.

### Dissemination plans {31a}

Trial findings will be shared through peer-reviewed publications, scientific conference presentations, and reports and dissemination events targeting relevant stakeholders. Results will also be communicated directly to study participants and the communities involved.

## Discussion

The SEED trial builds on prior evidence showing that Self-Help Plus (SH+), a brief, group-based psychological intervention developed by the World Health Organization, can reduce psychological distress among conflict-affected adults. In an earlier cluster-randomized trial conducted among South Sudanese refugee women in Rhino Refugee Settlement, Uganda [14], SH+ achieved short-term reductions in psychological distress at 3 months. However, questions remain about the persistence of these effects and their intergenerational implications for children’s development.

This study extends that evidence base in three important ways. First, by following participants for 12 months, it examines whether improvements in maternal mental health are sustained over time—a critical test of the durability and long-term value of scalable, low-intensity interventions in humanitarian contexts. Second, the study assesses whether these changes translate into measurable improvements in children’s psychosocial wellbeing and development. Third, it assesses parenting practices as a potential pathway linking changes in maternal distress to child outcomes. In addition, the trial includes prespecified exploratory measures of socio-behavioral and cognitive skills to generate hypotheses about mechanisms.

By combining measures of mental health and child development, the SEED trial addresses a major evidence gap: the lack of rigorous, longitudinal studies evaluating how psychological interventions can influence both individual and intergenerational outcomes in humanitarian settings. The design integrates cluster randomization for causal inference with field-appropriate behavioral and developmental assessments, balancing internal validity and ecological realism. The use of validated, internationally recognized instruments for both maternal and child outcomes ensures data quality and comparability with the wider literature. Furthermore, by collecting both parent- and child-reported data on child wellbeing (Kiddy-KINDL^R^), we incorporate a valuable multi-informant perspective on intergenerational outcomes.

A key scope consideration is the role of fathers. Intergenerational socialization occurs within families, and fathers (and other caregivers) can shape children’s preferences, behaviors, and wellbeing. The present trial, however, targets mothers and does not directly intervene with—or systematically measure outcomes for—fathers. As a result, our inferences focus on maternal pathways; any household-level spillovers that involve fathers cannot be isolated with these data. This design choice reflects the study’s primary aim and operational constraints in this setting, but it also identifies an important direction for future research: testing paternal and two-caregiver pathways, and examining whether combined or family-based approaches amplify or modify effects.

The findings from this trial will inform humanitarian policy and future research. Evidence of lasting and intergenerational benefits would provide a powerful rationale for scaling up SH+ as a cost-effective approach to build resilience in crisis-affected families. However, if the effects fade over time or do not extend to children, this would also be an important finding suggesting that a brief intervention like SH+ may not be enough on its own to produce long-term change. This would guide the next phase of research, pointing towards the need to test additional support such as follow-up “booster” sessions to maintain maternal gains, or combining SH+ with specific parenting programs to better support child development.

## Trial status

The protocol was first registered on ClinicalTrials.gov on 11 July 2025. This manuscript refers to protocol version 2. Manuscript submission occurred prior to recruitment and baseline data collection. Recruitment and baseline data collection were completed on 12 December 2025. Delivery of SH+ is ongoing. The final 12-month follow-up assessments are expected to be completed in February 2027.

## Supplementary Information

Supplementary Material 1: Table S1. Sociodemographic variables (SEED trial)

Supplementary Material 2: Table S2. Exploratory outcomes of the SEED trial

## Data Availability

All data and materials will be openly available on the Open Science Framework (OSF) website at this link: [https://osf.io/46qhb/?view_only=82598d792f304ee692fd636c94d5e7eb].

## Abbreviations

AAQ-II: Acceptance and Action Questionnaire-II
ACT: Acceptance and Commitment Therapy
APQ: Alabama Parenting Questionnaire
cRCT: Cluster-randomized controlled trial
EUC: Enhanced Usual Care
FIES: Food Insecurity Experience Scale
GAD-7: Generalized Anxiety Disorder-7
GDPR: General Data Protection Regulation
HAZ: Height-for-age *Z*-score
HDDS: Household Dietary Diversity Score
ICC: Intracluster correlation coefficient
ITT: Intention-to-treat
K6: Kessler-6 Psychological Distress Scale
KAB-REC: Kabale University Research Ethics Committee
LMM: Linear mixed-effects model
MAR: Missing-at-random
MCID: Minimal clinically important difference
MHPSS: Mental health and psychosocial support
MICS6: Multiple Indicator Cluster Surveys, round 6
MINI: Mini International Neuropsychiatric Interview
OPM: Office of the Prime Minister (Uganda)
OSF: Open Science Framework
PCL-C: PTSD Checklist - Civilian Version
PHQ-9: Patient Health Questionnaire-9
PSC-17: Pediatric Symptom Checklist-17
PSS-4: Perceived Stress Scale-4
PTSD: Post-traumatic stress disorder
RWC: Refugee Welfare Council
SEED: Self-Help Plus to Enhance Early Development
SH+: Self-Help Plus
SNOSE: Sequentially Numbered, Opaque, Sealed Envelopes
TOSA: Test of Spatial Assembly
ToT: Training of Trainers
TSC: Trial Steering Committee
UNCST: Uganda National Council for Science and Technology
UNHCR: United Nations High Commissioner for Refugees
WHO-5: WHO-5 Well-Being Index
WHODAS 2.0: WHO Disability Assessment Schedule 2.0
