# Supplementary material for: Self-Help Plus for refugee mothers in Rhino Refugee Settlement, Uganda (SEED): study protocol for a cluster-randomized controlled trial assessing intergenerational effects on preschool-aged children
Source: Trials. 2026 Feb 17;27:173. doi: 10.1186/s13063-026-09546-1 (PMC12930577; doi:10.1186/s13063-026-09546-1)
Supplement: Supplementary file 1 — Supplementary Material 1: Table S1. Sociodemographic variables (SEED trial) [file 13063_2026_9546_MOESM1_ESM.docx]

| **Supplementary Table S1:** Sociodemographic variables (SEED trial) | | | | | |
| --- | --- | --- | --- | --- | --- |
|  | **Description** | **Choice options** | **Measurement timepoint(s)** | | |
|  |  |  | **T0** | **T1** | **T2** |
| Mother age | “What is your age (in years)?” | (Integer) | X |  |  |
|  | “What is your birthday (month/year)?” | (Date) | X |  |  |
| Mother education | “What is your highest level of education? If you choose ‘some primary school’ below or ‘some secondary/high school’, please indicate ‘up to which class’ you attended.” | - No formal schooling - Some primary school - Completed primary school - Some secondary/high school - Completed secondary/high school - Some Technical Institute Training - Completed Technical Institute Training - Post-secondary qualifications other than university (e.g. Diploma) - Some University - University degree (e.g. Bachelor or Master) - Postgraduate education (PhD or higher) - (Doesn't know) - (No answer) | X |  |  |
| Marital status | “What is your relationship status?” | - Married and living together - Married but not living together - Being in a permanent relationship but not married - Separated or divorced - Widowed - (Doesn't know) - (No answer) | X |  |  |
| Household size | “How many people are currently living together with you in your household?” | - 1 - 2 - 3 - 4 - 5 - 6 - 7 - 8 - 9 - 10 - >10 | X |  |  |
| Number of adults living in the household | "How many adults (age >18) live in your household, *including you*?" | - Only me - 2 - 3 - 4 - 5 - 6 - 7 - 8 - 9 - 10 - >10 | X |  |  |
| Child age | Accurate birth dates are often unavailable in this refugee population, since mothers usually know only the birth year, and camp authorities default to assigning the 1st of January of that year (or their year of arrival in the camp) as the official birthday. To establish an accurate age estimate, we follow a three-step process:   1. Maternal report (“How old do you believe your child is?”) 2. Document check: Examine any available paperwork (birth certificate^[[1]](#footnote-1)^, camp ID, etc.). 3. Expert review: A trained research assistant determines whether the mother’s account and the documents align. If anything is unclear (for example because stunting can make children appear younger) the assistant seeks a second opinion from another team member.   The research assistant’s judgement, taking into account the consistency of all sources and the child’s appearance, is considered final (“To the interviewer: What is your expert assessment of the child’s age, in years?”). | (Integer) | X |  |  |
| Child sex | “Is your child a:...”. | - Girl - Boy - Prefer not to say | X |  |  |
| Time since displacement | “What year/month were you displaced from your area of origin?” | (Date) | X |  |  |
| Time since arrival in the camp | “What year/month did you come to Rhino Camp?” | (Date) | X |  |  |
| War exposure | We assess war‐exposure with a version of the Harvard Trauma Questionnaire (HTQ; Mollica et al., 1992) that Tol and colleagues adapted and validated for South-Sudanese refugee women in Rhino Camp, Uganda  [(Tol et al., 2020)](https://www.zotero.org/google-docs/?09FEoB). The module contains 24 Yes/No items, each asking whether the participant has personally experienced one of 24 distinct war-related events. | (24 Yes/No items) | X |  |  |
| Socio-economic status | “How much does your household earn (all money the family members earn including salary and side business income) in Ugandan Shillings per month?” | - 0 - 24,000 UGX - 25,000 - 49,000 UGX - 50,000 - 74,000 UGX - 75,000 - 99,000 UGX - 100,000 - 124,000 UGX - More than 150,000 UGX - (Doesn't know) - (No answer) | X | X | X |
|  | “Does your household have any of the following items?” | - Water tank - Table - Bed - Seed store - Non-mobile telephone - Mobile telephone - Smartphone - Electricity - Bicycle - (Doesn't know) - (No answer) | X | X | X |
| Vulnerability category | “What vulnerability category have you been assigned to in the camp?” | - 1 - 2 - 3 - (Doesn’t know) - (No answer) | X |  |  |
| Farming experience | “Please rate your experience in the following farming activities.”  (Experience in seven farming domains is assessed: Preparing the soil, Planting different kinds of vegetables, Harvesting, Managing and conserving water, Managing pests and diseases, Managing and storing crops post-harvest, and Marketing and selling crops.) | \| - Not experienced at all \| \| --- \| \| - Somewhat unexperienced \| \| - Moderately experienced \| \| - Experienced \| \| - Highly experienced \| | X | X | X |
| Exposure to natural hazards and environmental stressors | “Which of the following events occurred in your vicinity [baseline: during the last 12 months; follow-ups (T1/T2): since the last interview]? Select all that apply” | Events (select all that apply): Extreme heat; Extreme dryness; Heavy rainfall; Flooding; Wildfire; Severe storm; Volcanic eruptions.  If selected, impact rating:   - Slightly - Moderately - Significantly - Severely - (Doesn’t know). | X | X | X |
| Participation in other interventions/livelihood programs | “[Since you arrived in Rhino Camp / In the past 3 months / In the past 9 months], have you participated in any livelihood program besides general assistance (such as cash-for-work, vocational training, farming groups, etc.)?” | - Yes - No - Not sure | X | X | X |
|  | [If “Yes”:] “Please specify the name(s) or type(s) of program(s) you have participated in:” | (Text) |  |  |  |
| *Note:* T0, T1, and T2 refer to baseline, 3-month, and 12-month follow-ups, respectively. | | | | | |

**References**

Mollica, R. F., Caspi-Yavin, Y., Bollini, P., Truong, T., Tor, S., & Lavelle, J. (1992). The Harvard Trauma Questionnaire: Validating a Cross-Cultural Instrument for Measuring Torture, Trauma, and Posttraumatic Stress Disorder in Indochinese Refugees. *The Journal of Nervous and Mental Disease*, *180*(2). https://journals.lww.com/jonmd/Fulltext/1992/02000/The_Harvard_Trauma_Questionnaire__Validating_a.8.aspx

Norwegian Research Council. (2024). *Right from the start: Birth registration in Rhino Camp*. https://www.nrc.no/globalassets/pdf/briefing-notes/right-from-the-start/birth-registration-in-rhino-camp.pdf

Tol, W. A., Leku, M. R., Lakin, D. P., Carswell, K., Augustinavicius, J., Adaku, A., Au, T. M., Brown, F. L., Bryant, R. A., Garcia-Moreno, C., Musci, R. J., Ventevogel, P., White, R. G., & van Ommeren, M. (2020). Guided self-help to reduce psychological distress in South Sudanese female refugees in Uganda: A cluster randomised trial. *The Lancet Global Health*, *8*(2), e254–e263. https://doi.org/10.1016/S2214-109X(19)30504-2

1. In late 2023, the Office of the Prime Minister (OPM) and the Norwegian Refugee Council (NRC) issued birth certificates to all children under 5 living in Rhino Camp (Norwegian Research Council, 2024). [↑](#footnote-ref-1)
