# Supplementary material for: Self-Help Plus for refugee mothers in Rhino Refugee Settlement, Uganda (SEED): study protocol for a cluster-randomized controlled trial assessing intergenerational effects on preschool-aged children
Source: Trials. 2026 Feb 17;27:173. doi: 10.1186/s13063-026-09546-1 (PMC12930577; doi:10.1186/s13063-026-09546-1)
Supplement: Supplementary file 2 — Supplementary Material 2: Table S2. Exploratory outcomes of the SEED trial [file 13063_2026_9546_MOESM2_ESM.docx]

| **Supplementary Table S2.** Exploratory outcomes of the SEED trial | | | | | | |
| --- | --- | --- | --- | --- | --- | --- |
| **Outcome** | **Dimension** | **Measure** | **Measure Description** | **Assessment time points** | | |
|  |  |  |  | **T0** | **T1** | **T2** |
| Socio-behavioral skills | Patience | Investment task (1) | Participating mothers and their children will engage in a set of incentivized economic games to measure socio-behavioral skills, modeled on the approach of Chowdhury and colleagues (2). For mothers, one game will be selected at random for payout to minimize endowment effects; children will receive payouts for all games completed. Patience will be assessed through an investment task. Children will receive one token and choose whether to exchange it immediately for sweets or invest it for two tokens—and twice the amount of candy—at the end of the assessment. Mothers will receive five tokens, each worth 500 UGX (approximately €0.10), and will decide how many to keep versus invest. Invested tokens are doubled and paid out the following week. The number of tokens invested is the measure of patience. | X |  | X |
|  | Social preferences | Social preference task (adapted) (3,4) | To measure social preferences, we will ask participants to make a series of binary choices between two payoff distributions. In each decision, one option always distributes rewards equally (1:1), while the other provides an asymmetric allocation. Across three games, we manipulate the asymmetric option to assess different social preferences. In the prosocial game, the alternative payoff is (1:0), measuring basic prosociality. In the envy game, the alternative payoff is (1:2), capturing the willingness to benefit the partner at no personal cost. In the sharing game, the alternative payoff is (2:0), assessing advantageous inequality aversion. In the efficiency game, the alternative payoff is (2:3), where the total payoff is maximized, allowing us to measure a preference for efficiency over equality.  Both mothers and children will participate in the game, with the order of the games randomized. Each participant will play two iterations: one where the partner is described as living in their village and another where the partner is described as living in a distant village within the camp. | X |  | X |
|  | Risk | “Bomb” risk elicitation task (BRAT) (adapted) (5) | Risk-taking will be measured using an adapted version of the “bomb task.” In this task, participants will be presented with a set of boxes—five for mothers and three for children. One of these boxes contains an illustration of a crocodile, while the remaining boxes each contain a token. Participants decide how many boxes they want to open, knowing that finding a token allows them to keep it, whereas uncovering the crocodile results in winning zero tokens for that round. The number of boxes a participant chooses to open serves as the measure of risk-taking. | X |  | X |
| Nutrition | Food security | FIES (6,7) | The FIES a questionnaire consisting of 8 Yes/No questions that capture an individual’s access to adequate food. It assesses a range of food-insecure experiences and behaviors, from worrying about obtaining food to going hungry due to a lack of resources. Its standardized methodology allows for valid cross-cultural comparisons, making it a key tool for global food security monitoring. | X | X | X |
|  | Dietary diversity | Household Dietary Diversity Score (HDDS) (8) | The HDDS is a 12-item questionnaire that assesses household-level dietary diversity by summing the number of unique food groups consumed over the previous 24-hour recall period. As a key indicator of diet quality and micronutrient adequacy, the HDDS provides a validated proxy for a household’s economic access to a varied and nutritious diet, making it particularly relevant for evaluating food security interventions in vulnerable populations (9). | X | X | X |
|  | Child malnutrition | Height-for-age z-scores (HAZ) (10,11) | HAZ is a standardized indicator of linear growth and a primary measure of chronic malnutrition (stunting) in children. It reflects the long-term cumulative effects of nutritional and health conditions during early childhood. HAZ will be calculated for each child by comparing their measured height and weight to the median value for healthy children of the same age and sex, using the World Health Organization (WHO) Child Growth Standards as the reference population. | X |  | X |
| Cognitive skills (children) | Mathematics ability | Free counting (12) | In the free counting task, the children are asked to count as high as they can without error. | X |  | X |
|  |  | Give-n (12) | In the Give-n task, children are asked to give a certain number of discs (e.g., Could you give the puppet three discs to play with?) from a group of discs placed in front of the child. | X |  | X |
|  |  | Number comparison (12) | In the number comparison task, children are shown a number and asked relational questions (e.g., “What comes after 4?”). | X |  | X |
|  |  | Addition/subtraction (12) | In the addition and subtraction task, children are asked to complete a sequence of both verbal and non-verbal arithmetic operations. Initially, they engage in non-verbal addition and subtraction trials, followed by verbal trials. In the addition task (e.g. 1+1), the researcher places a disc on a cardboard in full view of the child. This disc is then hidden under a cover. The researcher then slides another disc under a fabric. Next, the researcher places two discs on the other cardboard in front of the child and lifts the cover to show the two discs on the cardboard and says, “Look, yours is just like mine.” This demonstration is then presented to the child again following the same procedure, but this time the child is asked to place the appropriate number of discs on the cardboard after being shown by the researcher. A verbal response is not expected. If the child has placed the wrong number of discs on the cardboard, the answer is corrected, and the researcher repeats the same procedure. The same procedure is followed as for the subtraction task (e.g., 2 - 1), but in this case the disk is removed from under the cover. In verbal addition/subtraction, the procedure remains the same; however, instead of physically placing the corresponding number of discs in front of them to represent the outcome, children are instructed to articulate the result verbally. | X |  | X |
|  | Spatial ability | 2-D TOSA (12) | In the 2D-TOSA task, children are asked to copy geometric designs, presented via a flat image on a card, using felt shapes. Performance is scored based on the accuracy of adjacent pieces, horizontal and vertical direction, and relative position of the pieces in their construction of the design. | X |  | X |
|  | Theory of mind | Surprise outcome (13) | In the surprise outcome task, children observe a story created using two dolls. One of the dolls (e.g., Sally) places a marble in her toy box. Then, Sally leaves the environment. Meanwhile, the other doll (e.g., Anne) takes the marble from Sally’s toy box and places it in her own. Anne returns and the researcher asks the child where Sally will look for her marble when Anne returns. After this false belief question, the reason for the given answer (“Why?”) is asked. | X |  | X |
|  |  | Surprise content (14) | In the surprise content task, the researcher asks the children what they believe is inside a candy box. After the child’s answer, it is shown that the candy box actually contains colored pencils. After these unexpected contents are placed in the box, the children are asked what they thought was inside the box before it was opened and what their friends would think was inside the box before it was opened. After these false belief questions, the reason for the given answer (‘Why?’) is asked. | X |  | X |
|  | Language ability | TIFALDI (15) | In the language task, children are asked to find the picture that matches the word the researcher said (e.g., television) among four different options. The pictures are shown to the children from a booklet with four pictures on each page. | X |  | X |
| Social capital | Group membership and participation |  | Social capital will be measured using a set of survey questions assessing group membership and participation in community activities. Respondents are asked whether they have been members of various groups in the past three months, including mothers’ groups, savings groups, religious groups, and political groups. Follow-up questions capture the frequency of participation in activities organized by these groups. | X | X | X |
|  | Trust |  | Trust will be measured using a series of questions assessing how much respondents trust different social groups, with responses ranging from 0 (not at all) to 4 (a lot). Trust is evaluated at multiple levels, including family, neighborhood, village, members of the respondent’s tribe in other villages, individuals from other tribes in the settlement, and people from Uganda more broadly. | X | X | X |
| The table presents the dimensions, measures, and descriptions of the exploratory outcomes, along with the time points at which assessments occur. Assessment time points: T0 (baseline), T1 (3 months post-intervention), and T2 (endline, 12 months post-intervention). Findings related to these exploratory outcomes will be reported in separate, dedicated publications. | | | | | | |

**References**

1. Angerer S, Bolvashenkova J, Glätzle-Rützler D, Lergetporer P, Sutter M. Children’s patience and school-track choices several years later: Linking experimental and field data. J Public Econ. 2023 Apr;220:104837.

2. Chowdhury S, Sutter M, Zimmermann KF. Economic Preferences across Generations and Family Clusters: A Large-Scale Experiment in a Developing Country. J Polit Econ. 2022 Sept 1;130(9):2361–410.

3. Fehr E, Bernhard H, Rockenbach B. Egalitarianism in young children. Nature. 2008 Aug;454(7208):1079–83.

4. Bauer M, Cassar A, Chytilová J, Henrich J. War’s enduring effects on the development of egalitarian motivations and in-group biases. Psychol Sci. 2014;25(1):47–57.

5. Crosetto P, Filippin A. The “bomb” risk elicitation task. J Risk Uncertain. 2013;47(1):31–65.

6. Cafiero C, Viviani S, Nord M. Food security measurement in a global context: The food insecurity experience scale. Measurement. 2018 Feb 1;116:146–52.

7. Cafiero C, Nord M, Viviani S, Del Grossi ME, Ballard T, Kepple A, et al. Methods for estimating comparable prevalence rates of food insecurity experienced by adults throughout the world. Rome Food Agric Organ U N. 2016;

8. Swindale A, Bilinsky P. Household dietary diversity score (HDDS) for measurement of household food access: indicator guide. Wash DC Food Nutr Tech Assist Proj Acad Educ Dev. 2006;

9. Ruel MT, Quisumbing AR, Balagamwala M. Nutrition-sensitive agriculture: What have we learned so far? Glob Food Secur. 2018 June;17:128–53.

10. WHO. WHO child growth standards: growth velocity based on weight, length and head circumference : methods and development [Internet]. Geneva: WHO Press; 2009 [cited 2024 Apr 19]. Available from: https://iris.who.int/handle/10665/44026

11. WHO. WHO child growth standards: Length/height-for-age, weight-for-age, weight-for-length, weight-for-height and body mass index-for-age; methods and development. Onis M de, editor. Geneva: WHO Press; 2006. 312 p. (WHO child growth standards).

12. Verdine BN, Golinkoff RM, Hirsh-Pasek K, Newcombe NS. Methods for longitudinal study of preschool spatial and mathematical skills. Monogr Soc Res Child Dev. 2017 Mar;82(1):31–55.

13. Baron-Cohen S, Leslie AM, Frith U. Does the autistic child have a “theory of mind” ? Cognition. 1985 Oct;21(1):37–46.

14. Kayasili BK, Acarlar F. The Development of Theory of Mind According to False Belief Performance of Children Ages 3 to 5. Educ Sci Theory Pract. 2011;11(4):1821–6.

15. Kazak Berument S, Güven AG. Turkish Expressive and Receptive Language Test: I. Standardization, Reliability and Validity Study of the Receptive Vocabulary Sub-Scale. Turk J Psychiatry. 2013;24(3):192–201.
